# Cross-fused renal ectopia associated with vesicoureteral reflux; a case report

**DOI:** 10.15171/jrip.2016.42

**Published:** 2016-08-03

**Authors:** Mitra Naseri

**Affiliations:** Pediatric Nephrology Department, Dr Sheikh Children Hospital, Mashhad University of Medical sciences, Mashhad, Iran

**Keywords:** Complications, Cross-fused renal ectopia, Renal ectopia, Urinary obstruction, Vesicoureteral reflux

## Abstract

Crossed renal ectopia is a rare urinary system anomaly which mostly is asymptomatic and is diagnosed incidentally. Urinary obstruction, infection, and neoplasia of the urinary system and nephrolithiasis are main complications of this anomaly. A 6-year-old boy admitted to the hospital with colicky abdominal pain and nausea. Abdominal examination revealed tenderness in right lower quadrant. Urine analysis and culture were normal. Kidney ultrasonography showed right kidney in pelvis cavity with no kidney tissue in left side. TC 99-DMSA scan demonstrated no radiotracer accumulation in the normal renal area. Radiotracer accumulation was seen in the pelvis area with a deviation to the left. Voiding cystoureterogram revealed right sided grade II vesicoureteral reflux. Severe urological anomalies in children may be asymptomatic or have nonspecific symptoms such as abdominal pain.

**Implication for health policy/practice/research/medical education:**

Significant urological anomalies in children frequently are asymptomatic. Ultrasonography suggested in children with recurrent abdominal pain or acute abdomen. Additionally, screning for urological anomalies by ultrasonography should be considered in prenatal period.

## Introduction

Renal anomalies include agenesis, multiple kidneys, renal ectopia, and fusion defects. Crossed renal ectopia is one of the rarest urinary system anomalies with a male predominance of 3:2. Most cases are asymptomatic and are diagnosed incidentally (1,2). Symptomatic cases present by abdominal pain, hematuria, fever, urinary tract infection (UTI), hypertension, renal failure, a palpable abdominal mass (1) or anorectal malformation (2) usually in the third or fourth decade of life.

This anomaly predisposes patients to urinary obstruction, infection, and neoplasia of the urinary system and nephrolithiasis (1,3-5). Crossed ectopia is always associated with abnormally located ureters, but the ureter or ureters usually enter the bladder at the normal position (6).

## Case Report

**Figure 1:**
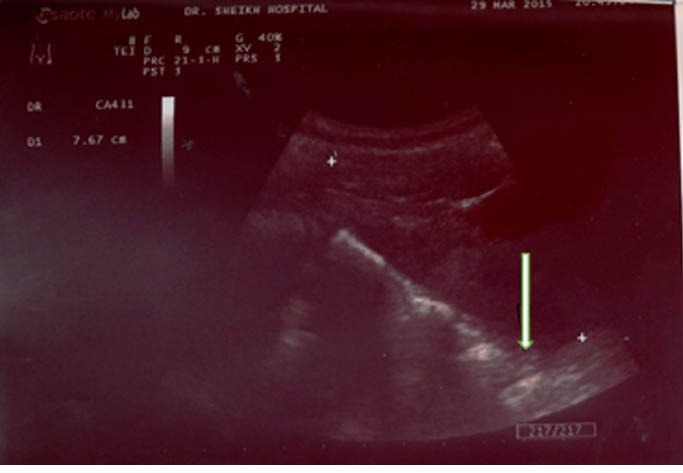
Abdominal ultrasonography which demonstrated right kidney in pelvis cavity with no kidney tissue in left side.

**Figure 2:**
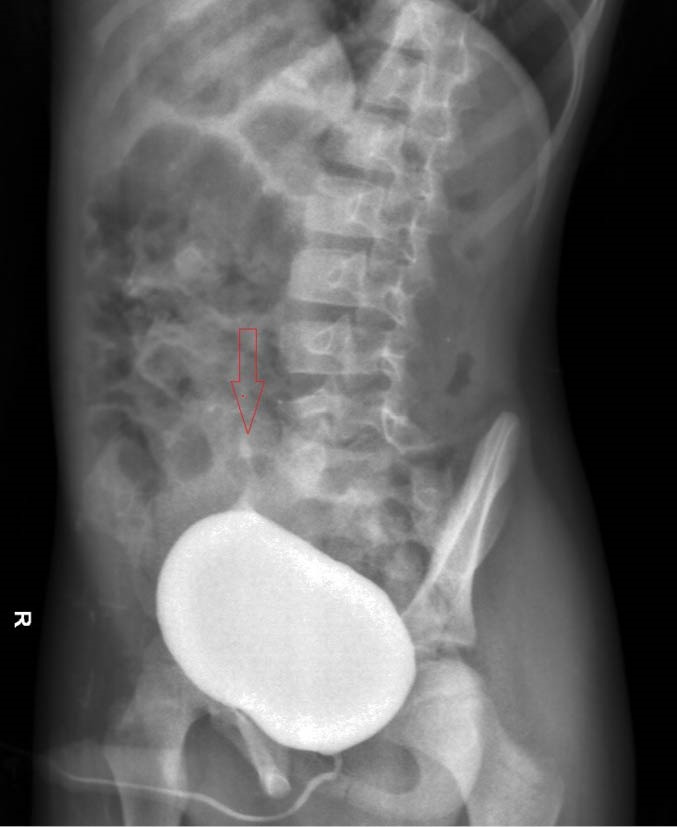
Voiding cystourethrography (VCUG) of the patient: grade II vesicoureteral reflux (VUR) in right kidney with normal bladder and urethra.

**Figure 3:**
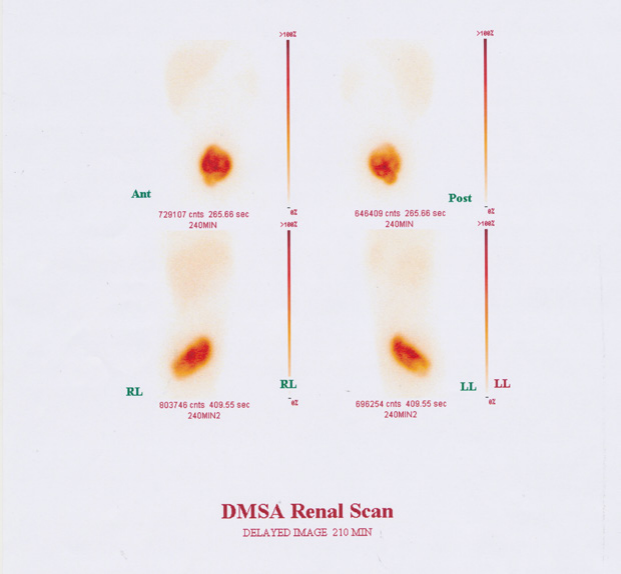
TC 99 DMSA scan in our patient which showed cross-fused ectopic kidneys localized in pelvic region.

A 6-year-old boy admitted in hospital with colicky abdominal pain and nausea from 5 days ago without urinary symptoms and no history of UTI in the past. Physical examination revealed mild abdominal tenderness in right lower quadrant without any rebound tenderness. Urine analysis and culture, and serum creatinine (Cr) level were normal (Cr=0.6 mg/dL). Abdominal ultrasonography showed right kidney in pelvis cavity with no kidney tissue in left side (Figure 1). In renal scintigram (TC 99-DMSA scan) there was no radiotracer accumulation in the normal renal area. Bizarre shape radiotracer accumulation was seen in the pelvis area slightly deviated to the left side that represented renal tissue. Measuring differential left and right renal tissues uptakes were not possible. The scan pattern suggested cross-fused ectopic kidneys localized in pelvic region (Figure 2). Voiding cystoureterogram (VCUG) was performed to rule out association with vesicoureteral reflux (VUR). It showed grade II right sided VUR with normal bladder and urethra (Figure 3).

## Discussion

Abnormal development of the ureteric bud and metanephric blastema during the fourth to eighth weeks of gestation seems result to crossed fused renal ectopia anomaly (2). The fusion abnormalities are clinically significant because half of cases complicated by obstruction, infections and nephrolithiasis. Left to right crossover occurs more frequently and the upper pole of the crossed ectopic kidney is fused to the lower pole of the normally located kidney in most instances. In our case a left to right cross-fused ectopia was detected with no complication (urinary obstruction, UTI or stone formation).

Although the exact incidence of this anomaly is not known because majority of patients are asymptomatic, a prevalence of 1:2000 to 1:7000 has been found in autopsy series (7,8). Boyan et al (1) reported two male adults with left-to-right inferior crossed renal ectopia with fusion; both had a history of chronic abdominal pain. Like our subject in cases reported by Solanki et al (2) VUR found in 4 of 6 patients cases. All of their patients were diagnosed in childhood period (up to age 10 years ). Two patients were asymptomatic, 2 cases were found during investigation for anorectal anomalies, one case presented by vomiting and abdominal pain and other had recurrent UTI. Although stone formation is one of the most common complication in patients with cross fused ectopia (3-5), our case had no evidence of nephrolithiasis in kidney-bladder ultrasonography.

Urological anomalies reported with cross-fused renal ectpoia are ureteropelvic junction (UPJ) obstruction, VUR and ectopic ureteroceles and rarely multicystic dysplastic kidney (7-9). There are no guidelines for the management of crossed fused renal ectopia. The fused renal units do not need to be separated and treatment depends on patient’s problems and associated complications.

As our subject did not need to surgical intervention, we did not recommend doing abdominal CT scan to be avoided radiation exposure. Abdominal CT scan can provide better information about degree of renal fusion.

Our case is interesting since TC99 DMSA scan indicated presence of severe fusion of renal tissues, because it was impossible to define the differential renal function of each kidneys due to overlapping of the kidneys’ tissues. Despite severe fusion that usually results to urinary stasis or obstruction, ultrasonography did not show evidence of obstructive uropathy or nephrolithiasis.

## Conclusion

Here we report a case of cross fused renal ectpoia who presented by symptoms mimicking acute abdomen. It was interesting while in addition to fused renal ectpoia, no evidence of urinary obstruction in kidney ultrasonography or stone formation was existed.

Severe urological anomalies in children may be asymptomatic or present with unspecific symptoms. In children who complain of chronic vague abdominal pain or those with acute or recurrent abdominal pain, imaging study for screening of renal anomalies or their complications (such as nephrolithiasis) is suggested.

## Acknowledgements

The author would like to appreciate Dr. Bostani (nuclear physician), Dr. Hebrani and Dr. Alamdaran (radiologist) for their advocated help.

## Conflicts of interest

The author declared no competing interests.

## Ethical considerations

Ethical issues (including plagiarism, data fabrication, double publication) have been completely observed by authors.

## Funding/Support

None.
